# Physicochemical and functional properties of rainbow trout (*Oncorhynchus mykiss*) hydrolysate

**DOI:** 10.1016/j.heliyon.2023.e17979

**Published:** 2023-07-07

**Authors:** Kristine Kvangarsnes, Egidijus Dauksas, Ignat Tolstorebrov, Turid Rustad, Martina Bartolomei, Ruoxian Xu, Carmen Lammi, Janna Cropotova

**Affiliations:** a Department of Biological Sciences Ålesund, Norwegian University of Science and Technology, Ålesund, Norway; b Department of Energy and Process Engineering, Norwegian University of Science and Technology, Varmeteknisk, 247, Gløshaugen, Trondheim, Norway; c Department of Biotechnology and Food Science, Kjemi 3, Gløshaugen, Sem Sælands vei 8, Trondheim, Norway; d Department of Pharmaceutical Sciences, Università degli Studi di Milano, Via Luigi Mangiagalli, 25, 20133, Milano, Italy

**Keywords:** Rainbow trout, Hydrolysates, Peptidomic investigation, DSC, Viscosity, Physicochemical parameters

## Abstract

Due to the continuous growth of the world population, there is an urgent need to find sustainable sources of high-quality protein. Fish side streams rich in essential nutrients and accounting for 60–70% of the whole fish, represent a sustainable source for recovery of valuable protein compounds. The present study aimed at extensive characterization of physicochemical, antioxidant and techno-functional properties of fish protein hydrolysate (FPH) obtained from farmed rainbow trout (*Oncorhynchus mykiss*). The FPH was produced from a minced rainbow trout raw material by enzymatic hydrolysis performed at 50 °C with addition of 0.05% w/w papain and 0.05% w/w bromelain. After inactivation of the proteases at 90 °C for 10 min, the content of the bioreactor was centrifuged, and the soluble protein fraction (FPH) was collected and freeze-dried. The total protein content of the FPH with 17.24% degree of hydrolysis was high (88.9%) and mainly represented by water-soluble proteins, while the lipid content was below 1%. In addition to the high protein content, trout hydrolysate had low protein oxidation values characterized by a relatively low total carbonyl content together with high amount of thiol groups (3.64 ± 0.31 and 20.7 ± 0.6 nmol/mg protein, respectively). No glass transition was detected in the differential scanning calorimetry (DSC) heat flow curves, suggesting lack of unfreezable solution formation in the FPH at freezing temperatures. The viscosity of FPH showed typical Newtonian behaviour. A peptidomic investigation (using HPLC-MS/MS technique) displayed chemical composition of the trout hydrolysate and identified peptide sequences which are present in the hydrolysate mixture, as well as proteins to which each peptide belongs to. In conclusion, it was suggested to use the obtained trout hydrolysate as a functional ingredient in the food and nutraceutical industry.

## 1 Introduction

The interest towards full utilization of fish catches including the side streams generated from fish processing plants to extract functional compounds for high value applications such as food ingredients and nutraceuticals, has increased to a large degree during the last decade [1]. Seafood side streams and by-products are rich in protein and essential nutrients such as polyunsaturated fatty acids, vitamins, and minerals. Nevertheless, today these valuable fractions are generally processed into low-value products such as fish meal, animal feed, or used as fertilizer or composted [1]. However, in the face of the world population growth expecting to reach 9 billion people by 2050, the global demand for food rich in high-quality protein has significantly increased. Norway is one of the leading producers of salmonids, accounting for about 2% of the global production (primarily Atlantic salmon (*Salmo salar*) and rainbow trout (*Oncorhynchus mykiss*)) [2]. Rainbow trout accounts approximately 97% of the global trout production set at 940,000 tonnes [2].

In the light of the new EU regulation on waste prevention and discards [3], the full utilization of the catch and conversion of fish side streams into valuable compounds would not only add value to the seafood processing sector but would also be able to decrease negative ecological impact of returning generated rest raw material and discards to the sea. Both the main product (fillet, etc.) and fish processing rest raw materials are good sources of high-quality protein compounds that may be used for human consumption [4]. Enzymatic hydrolysis is normally carried out under moderate conditions of pH and temperature [5]. The full utilization of fish raw material, including side-streams and by-products, would also help to increase the amount of high-quality protein ingredients, which may be used as a source of bioactive peptides with a number of functional and health-promoting properties [4].

The most commonly used methods for protein extraction from seafood raw material are the pH shift process, the surimi-method and enzymatic hydrolysis. During the pH shift extraction, a sample is homogenized in an acidic or alkaline solution followed by recovery of the proteins by centrifugation and isoelectric precipitation of the solubilized proteins in the supernatant. The surimi method for protein recovery consists of several washes of the raw material with cold water with or without 0.2% (w/v) NaCl, followed by refining and dewatering the slurry. However, the surimi process results in lower yields of protein than the pH shift method because a considerable amount of sarcoplasmic proteins are lost during the washing steps [6].

In enzymatic hydrolysis complex proteins are broken down into small peptides and free amino acids. Bioactive peptides are inactive within the parent protein but can be isolated by biochemical hydrolysis like enzymatic hydrolysis. The molecular mass is usually less than 6000 Da and usually contain 2-20 amino acids. Both the amino acid composition and the amino acid sequence affect the biopeptide activity [7,8]. Peptides containing between 5 and 16 amino acid residues have been found to possess antioxidant capacity [9].

Over the years fish protein hydrolysates have been reported to possess good functional properties like solubility in water, oil binding capacity, emulsifying, and foaming properties. The increase in solubility of the protein hydrolysates is due to the loss of secondary and tertiary structure, reduction in molecular size, and an increase in the number of polar and hydrophilic groups. Hydrolysates tend to be surface-active materials and promote oil-in-water emulsion as they comprise both hydrophilic and hydrophobic groups [10].

The techno-functional properties of the fish protein hydrolysate such as oil binding capacity, solubility, freezing and melting temperatures, as well as viscosity depend on both the type of raw material and the process parameters such as proteases used, pH, reaction temperature and duration of the hydrolysis [10,11]. The functional properties of protein hydrolysates also depend on intrinsic factors such as degree of hydrolysis, peptide size, shape, amino acid sequence and composition, and the ions present in the mixture. Among various parameters that determine the hydrolytic process, the degree of hydrolysis is used for monitoring the hydrolysis process. It can also be used as an indicator for comparing various protein hydrolysates [10,11] .

The present study aimed at extensive characterization of physicochemical, antioxidant and techno-functional properties of fish protein hydrolysate (FPH) obtained from farmed rainbow trout (*Oncorhynchus mykiss*). The study includes extensive characterization of techno-functional properties of rainbow trout hydrolysate together with its compositional analysis. The measurement of these parameters is crucial in ensuring the appropriate quality of the product.

## 2 Material and methods

### 2.1 Preparation of fish raw material

Head-on gutted rainbow trout (*Oncorhynchus mykiss*) was delivered from a local fish processing plant “*Hofseth AS*” (Ålesund, Norway) in January and August 2021, and handed over at slaughter day in insulated industrial boxes filled with ice. The fish were minced using a mincer with 4.5 mm hole size (Hobart A 200 N), divided into batches of 1 kg, and immediately frozen and stored at −80 °C until enzymatic hydrolysis.

### 2.2 Enzymatic hydrolysis

The enzymatic hydrolysis was performed 10 times, with an amount of 1 kg minced raw material each time. Hydrolysis experiments were performed in 4 L closed glass vessels placed in a water bath at 52 °C. Warm (50 °C) distilled water was added to fish mince in a 1:1 ratio. The mixture was stirred at 150 rpm with an overhead stirrer. When the temperature of the mixture was 50 °C, the enzymes Papain F6 and Bromelain 400 (Enzybel International S.A., Villers-le-Bouillet, Belgium) were added at levels of 0.05% (w/w) (0.1% in total). After 60 min of hydrolysis, bones were removed by filtering the hydrolysate through a sieve before the enzymes were inactivated by heating up to 90 °C for 10 min in a microwave oven. The mixture was cooled down up to 30 °C before being transferred to 1 L centrifugation bottles and then centrifuged at 4100 g at 4 °C for 30 min. The liquid fraction (lipids and water-soluble proteins) was separated from the insoluble fraction. The liquid fraction was placed in a separatory funnel and allowed to settle, and then separated into oil and water-soluble proteins. The water-soluble protein phase representing fish protein hydrolysate (FPH) was collected and dried in the laboratory vacuum freeze-dryer Alpha 2–4 LSC Plus, equipped with a LyoCube 4–8 chamber (Martin Christ GmbH, Germany) as described below**.**

### 2.3 Freeze-drying of protein hydrolysates

Vacuum-freeze drying was performed by the following procedure. The liquid samples were frozen at −24 ± 1.0 °C with the average thickness 0.010 ± 0.003 m. When the freezing process was completed and the temperature in the core of the sample reached the value of −24 °C. The samples were transferred into the laboratory vacuum freeze-dryer Alpha 2–4 LSC Plus, equipped with a LyoCube 4–8 chamber (Martin Christ GmbH, Germany). The drying pressure was set at 0.3 mbar. The temperature of the samples was slowly increased (0.4 °C min^−1^) from −24.0 to 10.0 °C during 48 h. As a result, the moisture content in the dried samples was in the range between 2.0 and 3.0% w.b. Then the dried samples were milled into powder by ball mill Pulverisette 6 (Fritsch, Germany) and vacuum-packed (50 mbar) in low density polyethylene bags.

### 2.4 Proximate analysis of raw material and hydrolysates

Total nitrogen was determined by using the Kjeldahl method, quantity of protein was calculated as 6.25 × N [12]. Content of water was determined gravimetrically after drying at 105 °C for 24 h. Ash content was determined by incineration to constant weight at 550 °C [13].

The extraction of lipids was performed according to the method of Bligh and Dyer [14]. Lipid content was calculated as g/100 g.

### 2.5 Water- and salt-soluble proteins in rainbow trout raw material

Sarcoplasmic (water-soluble) proteins and myofibrillar (salt-soluble) proteins were extracted from rainbow trout raw material as described by Anderson and Ravesi [15] and Licciardello et al. [16]. About two grams of minced muscle was homogenized in 40 ml of buffer (50 mM KH_2_PO_4_, pH7), and centrifuged at 4 °C for 20 min at 4100 g. The supernatant containing the sarcoplasmic proteins was decanted through glass wool and the volume was made up to 50 ml with buffer. To collect the myofibrillar proteins, the sediment was re-homogenized in 40 ml of buffer (50 mM KH_2_PO_4_, 0.6M KCl, pH7), centrifuged, decanted through glass wool, and volume was made up to 50 ml with buffer.

The protein content in the extract was determined in triplicate in suitable dilutions of both fractions by the method of Bradford [17]. Diluted color reagent (5 ml) was added to blank (distilled H_2_O), standards and samples, and absorbance at 595 nm was measured after 5 min using SpectraMax i3x Multi-Mode Plate Reader (Molecular Devices, LLC., San Jose, USA).

### 2.6 Degree of hydrolysis

The degree of hydrolysis (DH) was analyzed by formol titration as the proportion (%) of free amino groups with regard to the total nitrogen in the sample [18]. The total protein content results obtained from performing the Kjeldahl method were used to perform the calculation of the DH.

A sample of 1.5 g was weighed into a beaker and filled up to 50 g with distilled water. The pH was adjusted to 7.0 using 0.1 M NaOH and then 10 ml of 9% w/w formaldehyde with a pH of 8.5 was added into the beaker. The beaker was covered with aluminium foil and stirred for 5 min. For the titration, a TITROLINE 7000 automatic titrator (SI Analytics, Xylem Analytics Germany Sales GmbH & Co. KG, Germany), was used. The titrator was rinsed 3 times before starting the titration. Furthermore, the titration was set to pH 8.5 with stopping automatically when reaching a pH of 8.5. The samples were titrated with 0.1 M NaOH and the used amount of NaOH was recorded. To determine degree of hydrolysis, first the concentration of free amino groups was calculated according to the following formula (1):

(1) $$\frac{A\times B\times 14.007\times 100}{C\times 100}=\text{free}\;\text{amino}\;\text{groups}\;\left(\%\right)$$

where

A = ml NaOH used,
B = the concentration of the solution used for titration (0.1 M NaOH),
C = amount of sample (g).

Degree of hydrolysis was further determined by dividing the value of the free amino groups by the total amount of nitrogen calculated from the concentration of total protein (% protein divided by factor 6.25).

### 2.7 Determination of FPH solubility

The FPH solubility was determined at pH 2, 4, 6, 8 and 10. The peptide solubility was measured according to a method previously described [19,20]. Briefly, a solution of X μL of FPH at different pH, (100 − X) μL water, 95 μL 6% (w/w) NaOH in water, and 9.5 μL of active reagent (containing 0.6 M sodium citrate, 0.9 M sodium carbonate, and 0.07 M copper sulfate, 2.4 M NaOH, pH 10.6) was prepared. The reaction mixture was incubated for 15 min at room temperature (RT) and the absorbance was measured at 330 nm using the Synergy H1 plate reader.

### 2.8 Amino acid profile

About 50 mg of freeze-dried FPH was weighed into glass tubes, 1 ml 6 M HCl was added. The glass tubes placed into a heating cupboard for 24 h, at 105 °C. Samples were diluted 50 times using distilled water before filtering through 0.22 μm.

For the derivatization, 300 μl of the sample were transferred to a glass tube, containing 600 μl 0.4 M borate buffer (pH 9). 600 μl FMOC (9-fluorenylmethoxycarbonyl chloride, 15 mM in acetonitrile) was added, mixed, and then allowed to stand at room temperature for 10 min on an orbital shaker. After amino acid derivatization with FMOC, 600 μl ADAM (12 mM in acetone:water 1:1) was added.

Amino acids were analyzed using a Shimadzu Nexera XR HPLC system, equipped with a PDA detector (Shimadzu, USA). Separation of amino acids were carried out on a Restec ARC-18 column (10 mm × 2.1 mm) at 30 °C. The mobile phase was 0.1% formic acid with 20 mM ammonium formate and 0.1% formic acid with 10 mM ammonium formate in 90:10 acetonitrile water in gradient mode, with a flowrate of 0.35 ml min^−1^.

### 2.9 Total phenols – Folin-Ciocalteu assay

Deionized water, Folin-Ciocalteu reagent and the sample (protein extract from raw material and FPH) was mixed by vortexing. After 3 min, 1 ml of 20% Na_2_CO_3_ was added, and the volume was filled up to 10 ml by adding H_2_O. A calibration with concentrations ranging from 0.5 mM to 2 mM propyl gallate was prepared using the same reagents and procedure as water. The absorbance at 725 nm was read on SpectraMax i3x Multi-Mode Plate Reader (Molecular Devices, LLC., San Jose, USA) after 1 h of incubation at room temperature. For calculation, formula (2) was used.

(2) C=C1xV/m

where C = total phenolic content in mmol/g, C1 = concentration of propyl gallate established from the calibration curve, V = volume of extract in ml, and m = the weight of the sample in g.

### 2.10 Carbonyl groups

Carbonyl groups in the hydrolysate were determined using an immunoassay method developed by Buss et al. [21]. The ELISA kit, STA-310 Oxiselect TM was purchased from Cell Biolabs, Inc (San Diego, CA, USA). BSA standards (bovine serum albumin) and protein samples were adsorbed onto a 96-well plate at 4° overnight. The protein carbonyls present in the sample were derivatized with DNP hydrazine and probed with an anti-DNP antibody, followed by an HRP conjugated secondary antibody. The carbonyl contents in the samples were calculated by comparison against a standard curve of commercial reduced and oxidized BSA standards provided in the kit and expressed as nanomoles of carbonyl per milligram of protein (nmol mg^−1^ protein).

### 2.11 Thiol groups

Total thiol groups content was determined spectrophotometrically with Shimadzu UV-1800 UV/Visible Scanning Spectrophotometer (Shimadzu Europa GmbH, Germany) using the Ellman reagen (DTNB) [22]. To 100 μl of water soluble, and blanks (distilled water), 800 μl urea and 100 μl DTNB were added. Samples were mixed, incubated at room temperature for 30 min and centrifuged for 3 min at 11 300 g at room temperature. The absorbance was measured at 412 nm with a blank as reference. The thiol content was calculated using a molar extinction coefficient of 14,290 M^−1^ cm^−1^. The results are expressed as nmole/mg protein.

### 2.12 Color measurements

Color values of the protein hydrolysates were measured using a Minolta Chromometer Model CR 400 (Konica Minolta, Japan) calibrated on a white reference plate before use. L* (lightness), a* (redness) and b* (yellowness) were measured on the protein hydrolysates in triplicate at room temperature. The L*, a* and b* parameters of the CIELAB scale were measured according to the lab scale established by Commission Internationale de l’Éclairage [23].

### 2.13 Intrinsic fluorescence

The intrinsic fluorescence spectrum of FPH was obtained using a fluorescence spectrophotometer (Synergy H1, Biotek). The samples were diluted in phosphate-buffered saline (PBS, 10 mM, pH 7.0) in order to reach the equal concentration of 0.05 mg/ml and transferred in Greiner UV-Star® 96 well plates flat bottom clear cyclic olefin copolymer (COC) wells (cycloolefine). The excitation wavelength was set as 280 nm, while the excitation and emission slit widths were each set as 5 nm. The emission wavelength range was set up from 300 nm to 450 nm and the scanning speed was 10 nm/s.

### 2.14 Oil binding capacity (OBC)

The OBC was determined according to a method described previously [24] with some slight modifications. In details, 1 g of FPH sample was dispersed in 10 mL sunflower oil and vortexed for 1 min. The mixture was incubated at room temperature for 30 min and then centrifuged at 7000 g for 25 min at RT. The resulting supernatant was carefully decanted, and the tube containing the precipitation was weighed.

### 2.15 Mass spectrometry analysis (HPLC Chip ESI-MS/MS)

FPH sample was dissolved in 50 μL of 99% water and 1% ACN solution containing 0.1% formic acid. For the analysis, 2 μL of sample were injected in a nano-chromatographic system, HPLC-Chip (Agilent Palo Alto, CA, USA). The analysis was conducted on a SLIT mass spectrometer, using the same experimental conditions previously reported by Aiello et al. [25,26]. LC-MS/MS analysis was performed in data-dependent acquisition AutoMS(n)mode. Spectrum Mill Proteomics Workbench (Rev B.04.00, Agilent), consulting Oncorhynchus (56,588 entries) database downloaded from the Uniprot was used for the automated peptide identification from tandem mass spectra. The mass tolerance was set at 1.0 Da and 0.8 Da for MS1 and MS2, respectively. Auto-validation strategy of both peptide and protein polishing mode was performed.

### 2.16 DSC

The DSC analysis was done with a DSC Q2000 (TA Instruments, USA) equipped with a Liquid Nitrogen Cooling System (TA Instruments, USA). The temperature and cell constant calibration was done with indium. The heat capacity was calibrated with a sapphire in the range between −150.0 and 150.0 °C. Helium was chosen as a purge gas at 25 mL min^−1^, according to TA’s instrument recommendations. The reference sample was an empty hermetically sealed aluminium pan.

The samples with masses between 10 mg and 15 mg were placed into aluminium pans with hermetic lids. The pans were sealed with a Tzero® DSC Sample Encapsulation Press (TA Instruments, USA) and placed by an autosampler into the DSC cell.

Samples with a moisture content below 20.0% w. b. were cooled and equilibrated for 60 min at −150.0 °C; the cooling rate was 10 °C min^−1^. Then samples were heated to 150.0 °C with the heating rate of 10 °C min^−1^. Samples with a moisture content above 25.0% w.b. were cooled (10 °C min^−1^) and equilibrated at −150.0 °C for 60 min, then warmed up to −70.0 °C (5 °C min^−1^) and equilibrated for 5 min and cooled again to −150.0 °C (10 °C min^−1^) according to the sequence described by Tolstorebrov et al. [27]. The DSC scanning to +25.0 °C was performed at the same rate (10 °C min^−1^) as in the low moisture content samples.

The samples with different water content were obtained by dissolving the dried protein powder with distilled water. The moisture content of each sample was determined by the following method. After the DSC tests all the hermetic pans were punctured by a needle and dried at 105.0 °C until the constant weight. The water content was determined as a ratio between the pans weight difference before and after drying to % dry base.

#### 2.16.1 Determination of the glass transition, freezing and melting temperatures

The onset of ice melting, T_m_, and “end of freezing” was measured by analysing the DSC heating curve. The freezing point was estimated as a minimum value of the ice melting endothermic peak on the DSC heat flow curve in second derivation by temperature. The glass transition was determined with TA Universal Analysis 2000 version 4.5A software (TA instruments, USA). The onset and end of glass transition were measured by an extrapolation of the baselines to the intersection with the glass transition line. The inflection point of glass transition, T_i_, was found by derivation of the heat flow curve by temperature as described by Tolstorebrov et al. [28].

#### 2.16.2 Determination of unfreezable water

The amount of unfreezable water was detected by the analysis of the DSC melting curve. The DSC melting peaks were integrated with the sigmoidal tangent baseline function. The rough estimation of the mass of ice (I, kg) (ordinary method) was calculated by dividing the melting energy of ice in the fish, Ef_ish_, by the heat of fusion of pure ice L_ice_ [29]:

(3) $$I=\frac{E_{fish}}{L_{ice}}$$

L_ice_ = 333.5 kJ kg^−1^ at 0 °C. The enthalpy of ice melting depends on temperature of melting, T_m_. This leads to an underestimation of the ice quantity at temperatures below 0 °C. For correction purposes, the empirical equation suggested by Riedel [30] was used:

(4) $$L_{ice}=333.5+2.05*T_{m}-4.19*10^{-3}T_{m}^{2}$$

This equation describes the change of the heat of fusion with temperature accurately. In accordance with this, the ice fraction was estimated by Eq. (5) (Eq. (3) modified by Eq. (4) -modified method):

(5) $$x_{ice}=\frac{\int_{T}^{T_{f}}IdT}{\int_{T_{m}}^{T_{f}}IdT}$$

The dT was 0.02 °C. The amount of unfreezable water was obtained as the difference between the total water fraction in the product and the ice fraction.

### 2.17 Viscosity of FPH

The viscosity tests were performed on AR-G2 rheometer (TA instruments) equipped with cooling/heating module. The standard calibration procedure was done prior to tests. Pure distilled water was used for calibration; the deviation of viscosity did not exceed 0.1%. The experiment conditions were as follows: steady state flow test type with ramp of shear rate from 10 to 100 1/s with 5% tolerance. Standard steel plate of 40 mm was used as a moving surface. Viscosity of hydrolysates was determined at 25.0 °C. Samples with different water content were obtained by dissolving dried FPH powder in distilled water. The moisture content was determined by drying at 105.0 °C.

### 2.18 Statistical analysis

All results were expressed as the mean ± standard deviation (s.d.), where p-values <0.05 were considered to be significant. Statistical analyses were performed by one-way and two-way ANOVA followed by Tukey’s post-test (Graphpad Prism 9, GraphPad Software, La Jolla, CA, USA).

## 3 Results and discussion

### 3.1 Proximate analysis of raw material and hydrolysates

The proximate composition of rainbow trout raw material and the obtained FPH is shown in Table 1. Proximate parameters displayed similar values in regard to dry matter, ash, lipid and protein content in rainbow trout as reported in previous studies [5,31,32].

**Table 1:** Proximate composition of raw material and freeze-dried FPH.

	Raw material	Freeze-dried FPH
Dry matter (g/100 g)	46.51 ± 1.11	96.70 ± 0.23
Ash (g/100 g)	1.91 ± 0.29	7.37 ± 0.09
Lipid (g/100 g)	20.9 ± 0.8	<1
Protein (g/100 g)	23.7 ± 0.0	88.9 ± 0.1
Water-soluble proteins (%)	9.03 ± 0.01	∼100
Salt-soluble proteins (%)	2.66 ± 0.01	–

The high moisture and lipid content are in agreement with other studies characterizing rainbow trout raw material [5,[31], [32], [33]]. Protein constituted almost 25% of the fresh weight of the fish, suggesting that rainbow trout raw material might be a good source for protein extraction [5,33]. The proximate composition mostly depends on fish feed composition and growth conditions [34,35].

The obtained FPH had a relatively high protein content (88.9 ± 0.1 g/100 g w/w) compared to other studies reporting protein content of fish protein hydrolysates between 50 and 90% w/w [5,[36], [37], [38], [39]]. The high protein content demonstrates their potential to be used as protein ingredients for human consumption.

The ash content only showed small variations compared to other studies [32].

According to Table 1, the obtained trout hydrolysates had the following proximate parameters: 96.70 ± 0.23 dry matter, 88.9 ± 0.1 crude protein, and 7.37 ± 0.09 ash. Similar findings were found in the study of Kvangarsnes et al. [5] hydrolyzing heads of rainbow trout by using a mixture of papain and bromelain (0.05% w/w each) and Polat et al. [40] extracting FPH from fresh rainbow trout viscera and smoked trout trimmings using only papain. The trout hydrolysates obtained in our study were characterized with a very low lipid content (<1% w/w), which is very good indicator for oxidative stability of the product.

The raw material contained 9.03 ± 0.01% water soluble proteins, and 2.66 ± 0.01 salt soluble proteins. The solubility of both water-soluble and salt soluble proteins have been shown to decrease when the raw material undergo protein oxidation [5,41]. The obtained solubility of proteins in our study is higher compared to the solubility of proteins from rainbow trout heads (5.58 ± 0.16%) which can indicate a high quality of raw material [5]. In the produced FPH, 100% of the proteins was soluble, which indicate a high potential for the obtained FPH.

### 3.2 Quality characteristics of trout hydrolysates

The quality characteristics of FPH obtained from trout raw material is shown in Table 2.

**Table 2:** Quality characteristics of raw material and trout hydrolysates. Means and standard deviations are shown, n = 3.

Quality parameter	Raw material	FFP
Degree of hydrolysis (%)	–	17.24 ± 0.73
Total phenols (mmol/g)	0.008 ± 0.001	0.012 ± 0.002
Carbonyl groups (nmol/mg protein)	2.61 ± 0.56	3.64 ± 0.31
Thiol groups (nmol/mg protein)	45.3 ± 3.3	20.7 ± 0.6
Color parameters		
«Lightness, L»	53.47 ± 0.44	90.60 ± 0.21
«Yellowness, b»	29.80 ± 1.16	13.47 ± 0.31
«Redness, a»	16.73 ± 2.21	−0.43 ± 0.06

The degree of hydrolysis (DH) of trout hydrolysates was 17.2 ± 0.7% which is in agreement with other studies using the plant-based enzymes papain or/and bromelain for enzymatic hydrolysis of fish raw material [5,42,43]. The DH is mainly affected by the type of proteases used during the hydrolysis process, as well as temperature, pH, and duration of hydrolysis [44]. According to Dauksas et al. [45], DH levels ranging between 4 and 40 give the highest risk of bitterness in hydrolysates. However, increasing the degree of hydrolysis generally increase the number of short-chain peptides, resulting in a hydrolysate with higher solubility and bioactive and functional properties [42,46].

In addition to bitterness and functional and bioactive properties, color is one of the most important sensory parameters of the hydrolysate, affecting the overall product acceptance [47]. Color is the first sensory attribute assessed by the consumer. Moreover, for the successful commercialization of this ingredient, it is particularly important to know how the color of the hydrolysate can affect the color of the final food product in which it is incorporated. Hydrolysates from rainbow trout and herring raw material have been reported to be darker and have a more undesirable yellow color than hydrolysates obtained from chicken raw material [48,49]. However, the trout hydrolysate obtained in the present study has high lightness values (90.60 ± 0.21) together with negative values of redness (−0.43 ± 0.06) and average positive values of yellowness (13.47 ± 0.31), suggesting a pleasant yellow-whitish color of the product. The average positive values of yellowness can be explained by the natural orange-reddish pigment astaxanthin which is found in the flesh of rainbow trout together with lipids.

The carbonyl groups (2.61 ± 0.56) and thiol groups (45.3 ± 3.3) in raw materials shows that the starting material is of high quality. Trout hydrolysate obtained in the present study had low amount of total carbonyls (3.64 ± 0.31 nmol/mg protein) and relatively high amount of thiol groups (20.7 ± 0.6 nmol/mg protein), suggesting low level of protein oxidation. Normally, the oxidative stress results in the increase in carbonyl groups and the formation of disulphide bonds, leading to a reduction in number of thiol groups [50]. The increase of carbonyl groups occurs during the oxidation of amino acid side chains, and particularly the side chains of proline, asparagine, lysine, arginine, and threonine forming carbonyl residues due to metal-catalysed reactions also involving the oxidation of haemoglobin and myoglobin [50]. Thus, the low level of carbonyls in the present study can be explained by the use of fresh and previously gutted and bled fish raw material. In addition, rainbow trout is well known to contain natural antioxidant – astaxanthin, which makes it possible to retard lipid oxidation caused by free radicals in the fish flesh leading, which can further lead to protein oxidation. Astaxanthin is one of most powerful antioxidants in marine organisms which can effectively decrease oxidative stress in rainbow trout raw material [51]. Phenolic compounds were relatively low in raw material of trout (0.008 ± 0.001 mmol/g). Fish is known to be a potential source of phenolic compounds which have antioxidant and antimicrobial properties. The amount of phenolic compounds in the FPH was 0.012 ± 0.002 mmol/g. During enzymatic hydrolysis, the phenolic compounds can be released from proteins, skin and bones, resulting in a higher amount of total phenols.

### 3.3 Amino acid composition of trout hydrolysates

FPH usually contain peptides consisting of 2–20 amino acids. These short peptides possess much stronger antioxidant capacity than free amino acids due to the increased stability of the resulting peptide radical [52]. However, since the biological activities of peptides depend on their amino acid composition and sequence [53], it is very important to study the amino acid composition of trout hydrolysates.

The amino acid profile of the trout hydrolysates obtained in our study is shown in Table 3. The most abundant amino acids in the trout hydrolysate are: Glycine (15.50%), Lysine (11.61%), Glutamic acid (11.15%), Proline/Methionine (10.83%), Tyrosine (8.00%), Aspartic acid (7.63%), Alanine (7.02%), and Leucine (7.67%). These results are in the same range as reported by Li et al. [32] and Kvangarsnes et al. [5]. Isoleucine and Leucine as well as Proline and Methionine are merged in our study.

**Table 3:** Amino acid composition of trout hydrolysate, % of amino acids.

Amino acid	%
Arginine	3.38
Serine	3.73
Aspartic acid	7.63
Glutamic acid	11.15
Threonine	4.21
Glycine	15.50
Alanine	7.02
Proline/Methionine	10.83
Valine	5.24
Phenylalanine	4.05
Isoleucine/Leucine	7.67
Lycine	11.61
Tyrosine	8.00

The trout hydrolysates analyzed in the study can be considered of high nutritional protein quality, according to daily requirements for adults [54,55] suggesting that an adult of 70 kg requires about 6 g of indispensable amino acids (IAA) on average per day. Thus, the obtained FPH can be used in a high number of food applications in the future as high-quality protein ingredient.

### 3.4 DSC results

Onset of ice melting (end of equilibrium freezing) was detected for sample with moisture content over 16.1% d.b. in the temperature range between −30.9 and −33.7 °C. The temperature is very similar to the onset of ice melting of rainbow trout muscles, which was reported previously (−30.8 ) [27]. The beginning of ice melting at DSC reflects the end of ice formation at equilibrium conditions, for example, at frozen storage. Thus, decreasing the storage temperature of the frozen hydrolysates below this value will not result in the subsequent ice formation.

The samples with the moisture content below 16.1% showed only glass transition, which varied in the range between −11.4 and 43.8 °C with respect to water content. The reported data for low-moisture content FPH (the origin and degree of hydrolysis was unknown) revealed the glass transition in the range between −30 and 41 °C, when the moisture content varied from 16.3 to 2.1% d.b. [56]. The FPH obtained from the horse mackerel showed glass transition in the range between −42.8 and 71.9 °C, when the water content varied from 20% to 0% respectively [57].

The freezing point of the FPH strongly depends on the dry matter and decreases together with decreasing the moisture content. The applied method of unfreezable water determination [28] revealed the presence of the unfreezable was in all the samples where is ice formation was detected. The ratio of unfrozen water to solids was calculated to be between 27.8 and 40%, Table 4.

**Table 4:** Thermal properties of FPH with respect to moisture content.

Water, % d.b.	Glass transition, °C	Melting energy, kJ/kg	Ice, %	Unfreezable water to solids, %	Freezing point, °C	T_m_, °C
1480.4	N/D	297.4	91.2	38.3	−0.66	−31.5
450.6		240.1	75.7	33.9	−1.86	−31.6
439.6		235	74.1	40.0	−1.74	−31.5
271.6		199.8	63.6	35.2	−3.01	−31.7
212.8		179.4	57.6	32.7	−4.29	−30.9
119.6		111.1	36.8	38.8	−7.45	−32.6
96.0		114.4	38.0	21.5	−7.32	−31.0
89.2		77.59	26.7	38.7	−10.71	−33.7
77.9		81.74	28.1	27.8	−10.39	−33.5
53.5		28.23	10.0	38.3	−16.6	−32.2
53.2		29.21	9.9	38.0	−16.98	−31.7
16.1	−11.4	N/D	N/D		N/D	N/D
15.5%	0.1					
12.0%	20.4					
8.7%	43.8					

**Fig. 1:**
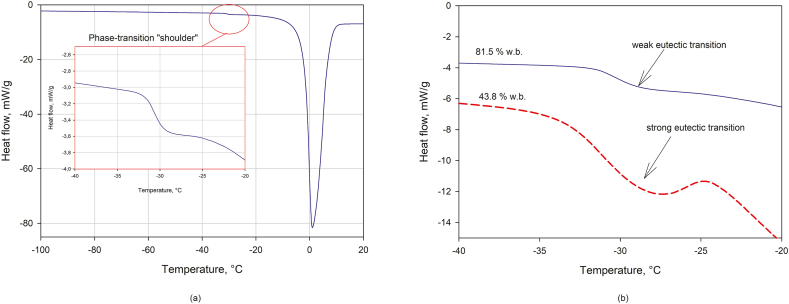
Determination of phase transitions in FPH with respect to moisture content.

The so-called unfreezable water forms the maximal freeze concentration, which undergoes the glass transition phenomenon with further decrease in temperature [58]. The glass transition occurs in the fish muscles in the relatively wide temperature range between −27 and −90 °C and it is well described in the literature [27,28,[59], [60], [61], [62], [63], [64]]. However, in this study the glass transition was not detected for FPH samples with a water content above 53.2% d.b. The absence of the glass transition can be explained by the analysis of the DSC melting curve, Fig. 1.

The melting shoulder was observed at vicinity of onset of ice melting, Fig. 1a. The shape of the shoulder (step change of the heat flow) is very similar to the glass transition. At the same time, the increase in the solid fraction in FPH lead to transformation of the “shoulder” to separate melting peak, Fig. 1b (dashed line). The second melting peak is common for saturated solutions of salts in the vicinity of the eutectic point. Free amino acids can form eutectic solutions with salts at certain temperatures below the freezing point of the solution. In addition, the presence of the eutectic melting peak was mentioned as an indicator of the low cryo-protective effect of some amino-acids [65]. The FPH is characterized by a medium degree of hydrolysis (17.24%), where a significant part of the proteins are presented by free amino acids, peptides and polypeptides. Thus, the presence of the second melting peak can be explained rather by eutectic point than the glass transition phenomenon. In this case, all the elements in the FPH solution are in the crystalline-solid state below the “end of freezing point” and the maximal freeze concentration was not formed. The DSC heat flow curves showed absence of the glass transition even at the glass transition temperature of water −142 °C, which indicates that the unfreezable solution was most likely not formed in FPH at freezing temperatures. The calculated difference between total water content and ice content, which is known as unfreezable water, was the result of the eutectic transition. Eutectic transition has less latent energy and influence significantly on the applied method of the unfreezable water determination.

### 3.5 Viscosity

The Herschel-Bulkley model was used to describe the viscosity alteration with respect to dry matter content, Table 5. The models can be described by eq. (6):

(6) $$\tau =\tau_{0}+k\gamma^{n}$$

where, τ is shear stress, Pa; $\tau_{0}$ – yield stress, Pa; k – consistency index, Pa*s^n^; γ – shear rate, s^−1^; n – flow index.

**Table 5:** Rheological properties of FPH with respect to dry matter content.

Dry matter, w.b., %	Parameters of Herschel-Bulkley model		
	Yield stress, Pa	Consistency index, Pa*s^n^	Flow index, -
5.86	0	0.0012 (0.0001)	0
17.4	0	0.0026 (0.0001)	0
45.1	0	0.0786 (0.006)	0
54.3	8.05 (0.4)	1.170 (0.08)	0
68.1	23.1 (0.8)	5.770 (0.05)	0

The viscosity is determined as a ratio between shear stress and shear rate. The FPH with dry matter below 45.1% w.b. showed typical Newtonian behaviour, which means that the viscosity is a constant value irrespective of the fluid flow velocity and it equals to the consistency index. The same behaviour is normal for water, oils and other fluids. Increasing the dry matter resulted in the subsequent increase of the consistency index, so the viscosity is increasing. The recent study of FPH from stone fish revealed that the viscosity was independent from flow velocity at low concentrations and varied in the range between 0.003 and 0.04 Pa*s (for concentration from 1.0% to 10% w/v respectively) [65].

The flow index was measured at 0 for all the sample, which means that the shear stress increases linearly with the shear rate. Dry matter content of 54.3%.w.b and above resulted in the appearance of Yield stress. The FPH showed Bingham Plastic Liquid Behaviour, where the stress should be applied to the fluids before it starts to flow. Thus, the viscosity will strongly depend on the flow velocity of the fluid. The FPH from stone fish revealed a consistency index n < 1 for high concentrations of dry matter in hydrolysates, which refers to pseudoplastic liquid [65]. However, both studies revealed shear thinning effect at high concentrations, which is in agreement with other studies on non-fish protein hydrolysates, soy protein hydrolysate [66] and milk protein hydrolysate [67].

### 3.6 Mass spectrometry analysis (HPLC Chip ESI-MS/MS)

The composition of the peptides of FPH hydrolysate was analyzed by HPLC-ESI-MS/MS. Table 5 lists the identified peptides from the most abundant proteins. Among these, twenty peptides were identified in the trout hydrolysate sample. The length of those peptides ranged from 13 to 32 amino acids with a molecular weight in the range 414.65–957.74 Da, and the 20% of the identified peptides belong to the Myosin protein. The molecular weight of the peptides (<1000 Da) is in line with the enzymatic activity of the two enzymes chosen for hydrolysis, as previously reported in other studies [68,69]. This mass range is advantageous for the search of bioactive peptides such as antioxidant peptides whose molecular weight usually ranges between 500 and 1000 Da [70]. Furthermore, the presence of low molecular weight peptides which have a higher content of polar residues than intact proteins, ensures greater solubility [71].

The identified peptides displayed a calculated isoelectric point (pI) in the range 3.5–10.16, a hydrophobicity in the range 13.04–42.19 kcal/mol and a net charge in the range −6 – +2. The wide range of ionic strengths is due to the variability of the aa present in the hydrolysate, like the abundance of lysine and glutamic acid, and contribute to the functional properties of the hydrolysate such as good solubility, water and old holding capacities [71], Table 6.

**Table 6:** Peptide sequence from trout protein (scores>10).

Sequence	*m*/*z* (z)[Da]	Intensity	Isoelectric point (pI)^a^	Hydrophobicity (Kcal/mol) a	Net Charge a	protein
(K)TEPGSLPEGKVK(I)	414.65 (3)	3.58E+06	6.70	22.34	0	Myosin motor domain-containing protein
(K)IRLESDGSLLDVDEDDVEK(A)	716.24 (3)	3.03E+07	3.5	37.88	−6	
(K)TELHFNHFAENSAFGIVPQPKSEDK(Q)	947.93 (3)	2.66E+07	5.21	30.88	−2	
(K)DLKRTKVLLADAQIMLDHMK(N)	780.22 (3)	5.18E+07	9.87	25.46	+1	
(K)QRPSSTTTDTGK(L)	426.82 (3)	7.05E+07	9.84	20.13	+1	Ankyrin_rpt-contain_dom domain-containing protein (Fragment)
(K)DCKKSRFSSDIVGPSDPQPDK(N)	788.63 (3)	8.43E+07	6.39	33.54	0	
(K)TPVESGASSAENRAADSTMTTSKPK(D)	841.5 (3)	3.60E+07	6.77	32.66	0	
(K)LCAEPVAESAKSEHAVTEESETK(D)	835.35 (3)	9.09E+07	4.08	39.44	−4	Peptidase_M24 domain-containing protein
(K)RGGITCFLKVKCEEEMINDTMK(L)	887.7 (3)	1.32E+07	6.30	29.25	0	
(K)QHIIDGEKTIIQNPTDQQRKDHEK(A)	957.74 (3)	9.08E+06	6.08	42.19	−1	SH3_10 domain-containing protein
(K)SEHEVQDAELRTLLQSSASRKTQK(K)	914.33 (3)	7.21E+07	7.61	35.42	0	
(K)IRCVEEKPVLSLPCVPHVAPPSNPK(A)	942.68 (3)	1.03E+07	8.27	22.51	+1	SH3 domain-containing protein
(K)EVIRLEKDPEMLK(A)	534.07 (3)	5.96E+06	4.52	25.23	−1	
(K)ALYTQYLQFKENEIPLKETEK(S)	862.73 (3)	3.52E+07	4.55	26.35	−1	SH3 domain-containing protein
(K)MSHKSAVANGGGPGNHAYLTNK(E)	737.71 (3)	3.28E+07	10.15	25.03	+2	
(K)AISLNLNNYEK(E)	426.82 (3)	1.73E+07	6.78	13.51	0	
(K)VSYECRVVSGKLVMGLDK(M)	681.33 (3)	1.06E+07	8.86	20.66	+1	Fibrillar collagen NC1 domain-containing protein
(K)IVESYNTVSVLGVSK(S)	797.89 (3)	3.96E+07	6.75	13.04	0	
(K)GSLGPFGVPGQVGPK(G)	466.27 (3)	3.60E+06	10.16	14.22	+1	Polymerase basic protein 2
(K)GLQGSPGPMGKEGDVGPLGDAGGPGSKGEK(G)	912.46 (3)	1.15E+07	4.47	42.61	−1	

a Calculated according to free PepDraw software (https://pepdraw.com).

### 3.7 Intrinsic fluorescence of FPH

**Fig. 2:**
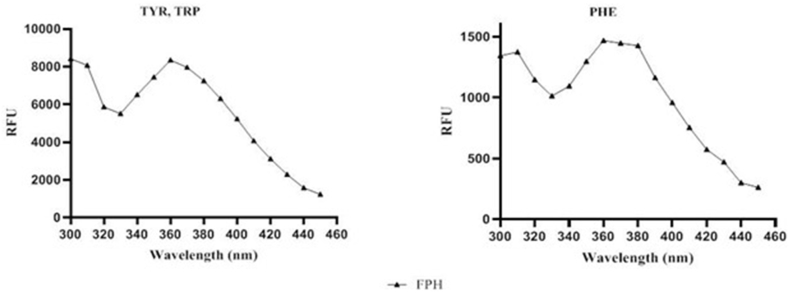
Intrinsic fluorescence signal detection of FPH.

Intrinsic fluorescence spectroscopy was used to detect the fluorescence due to the presence of tryptophan (Trp), tyrosine (Tyr) and phenylalanine (Phe), which are excited respectively at 280 and 250 nm. The graphs showed peaks at 310 and 360 nm, which indicate a shift towards a longer emission wavelength, demonstrating that the chromophores are exposed to an aqueous environment (Fig. 2).

### 3.8 Peptide solubility (PS) and oil binding capacity (OBC) of FPH

**Fig. 3:**
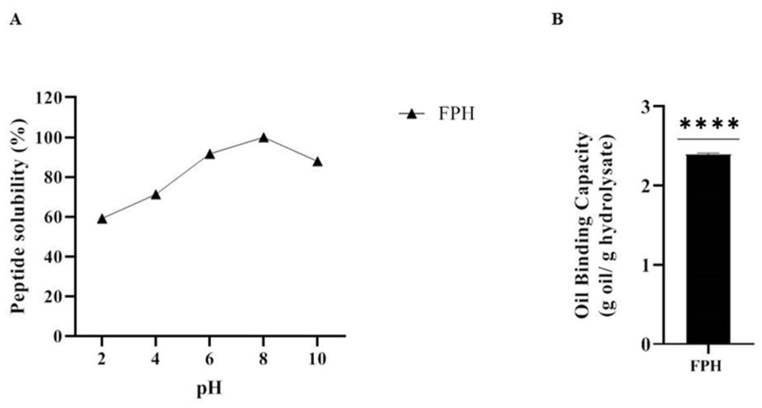
Peptide solubility (PS) (A) and oil binding capacity (OBC) (B) of FPH. Statistical analysis was performed by one sample *t*-test. (****) p < 0.0001.

The peptide pH-solubility profile of FPH sample is shown in Fig. 3A. The graph shows that the best solubility is found at alkaline pH, with the highest solubility at pH 8. Conversely, lower solubility has been observed in the pH range below 6. In detail, FPH reach 59.2 ± 2.03%, 71.19 ± 0.69%, 91.7 ± 0.7%, 100% and 87.91 ± 0.19% solubility at pH 2, 4, 6, 8 and 10, respectively (Fig. 3A). Peptide solubility is an important factor to consider, due to the impact on their functional properties.

The physical incorporation of oil or fat by the hydrolysate proteins was determined by the OBC study. In detail, the OBC for the FPH sample is 2.39 ± 0.015 g oil/g hydrolysate (Fig. 3B). During hydrolysis, proteins are converted to smaller peptides and amino acids which can increase the solubility of peptides, and oil binding capacity, as the smaller peptides or amino acids can better interact with water or oil molecules. The oil binding capacity is important in various food and industrial applications, such as production of emulsions, sauces, and coatings, and is also influencing the taste and functional characteristics of products.

## 4 Conclusion

FPH obtained from rainbow trout raw material has been shown to have good physicochemical and functional properties compared to those of well-established food proteins. By hydrolyzing fresh rainbow trout raw material with commercial enzymes papain and bromelain (0.05% w/w each), it was possible to produce FPH with high content of protein, low content of lipid and very low level of protein oxidation. FPH contains all essential amino acids and is especially a good source of lysine (11.61%). Hydrolysates reached the highest solubility at pH 8 and it clearly displayed oil binding ability. The presence of 20 different peptides were detected by peptidomic study, which allowed identification of the most abundant medium-chain peptides in the hydrolysate. In addition, the amount of phenolic compounds in the FPH increased significantly up to 0.012 ± 0.002 mmol/g compared to initial trout raw material due to their release from the fish flesh, skin and bones during enzymatic hydrolysis. The DSC analysis revealed the presence of the unfreezable water in the trout hydrolysate samples with water content above 53.2% dry base. The ratio of unfrozen water to solids was calculated in the range of 27.8–40.0% with respect to water content. The intrinsic fluorescence spectroscopy applied to FPH revealed peaks at 310 and 360 nm corresponding to tryptophan, tyrosine and phenylalanine shifted towards a longer emission wavelength, which indicated that the chromophores were exposed to an aqueous environment.

Due to the high physicochemical quality and amphiphilic nature of FPH determining their functional properties, it was suggested that rainbow trout hydrolysates may be used in a large number of food applications in the future.

## Author contribution statement

Kristine Kvangarsnes, Egidijus Dauksas, Martina Bartolomei, Ruoxian Xu: Performed the experiments; Analyzed and interpreted the data; Wrote the paper.

Ignat Tolstorebrov, Carmen Lammi: Performed the experiments; Analyzed and interpreted the data; Contributed reagents, materials, analysis tools or data; Wrote the paper.

Turid Rustad: Analyzed and interpreted the data; Contributed reagents, materials, analysis tools or data; Wrote the paper.

Janna Cropotova: Conceived and designed the experiments; Performed the experiments; Analyzed and interpreted the data; Contributed reagents, materials, analysis tools or data; Wrote the paper.

## Data availability statement

Data included in article/supp. material/referenced in article.

## Declaration of competing interest

The authors declare that they have no known competing financial interests or personal relationships that could have appeared to influence the work reported in this paper
